# Optoelectronic Properties of MAPbBr_3_ Perovskite Light-Emitting Diodes Using Anti-Solvent and PEDOT:PSS/PVK Double-Layer Hole Transport Layers

**DOI:** 10.3390/mi13122122

**Published:** 2022-11-30

**Authors:** Kai Zhang, Shisong Yu, Peng Tu, Xiangcheng Cai, Yuanming Zhou, Fei Mei

**Affiliations:** 1 School of Electrical and Electronic Engineering, Hubei University of Technology, Wuhan 430068, China; 2 School of Science, Hubei University of Technology, Wuhan 430068, China

**Keywords:** perovskite, additive, hole transport layer, poly(9-vinylcarbazole) (PVK)

## Abstract

Perovskite light-emitting diodes (PeLEDs) have attracted extensive attention due to their advantages such as low-temperature solution processing, high photoluminescence quantum efficiency, high color purity, tunable wavelength, and excellent carrier mobility. The hole transport layer plays an important role in the device’s performance. In this paper, the effect of anti-solvent (ethyl acetate) on the performance of PeLEDs was studied in order to determine the optimal anti-solvent condition. The effect of PEDOT:PSS/PVK double-layer hole transport layers on the optoelectronic properties of MAPbBr_3_ PeLEDs was investigated. The device with 8 mg/mL PVK produced the best results, with a maximum luminance of 5139 cd/m^2^ and a maximum current efficiency of 2.77 cd/A. Compared with the control device with PEDOT:PSS HTL, the maximum luminance of the device with 8 mg/mL PVK is increased by 2.02 times, and the maximum current efficiency is increased by 188%. The experimental results show that the addition of PVK helps to reduce the size of perovskite particles, contributing to the spatial confinement of excitons, and suppress the quenching of luminescence occurring at the interface between PEDOT:PSS and MAPbBr_3_, thereby enhancing the optoelectronic performance of PeLEDs. The results of this paper can provide a basis for the improvement and industrialization of PeLEDs.

## 1. Introduction

Light-emitting diodes (LEDs) are widely used in solid-state lighting and flat-panel displays [1,2,3,4,5]. Perovskite light-emitting diodes (PeLEDs) have attracted extensive attention due to their advantages, such as low-temperature solution processing, high photoluminescence quantum efficiency, high color purity, tunable wavelengths, and excellent carrier mobility [6,7,8,9,10,11,12,13,14]. The hole transport layer (HTL), which is crucial for hole injection, affects the number of holes transported into the emission layer (EML) region and correspondingly the radiative recombination of excitons [15,16,17,18]. Poly(3,4-ethylenedioxythiophene)-poly(styrenesulfonate) (PEDOT:PSS) is a widely used HTL, and the intercalation of PEDOT:PSS between ITO and perovskite EML can reduce the surface roughness of ITO and the hole injection barrier. However, due to the large energy level difference between PEDOT:PSS and perovskite, excitons can be easily quenched through nonradiative energy transfer and/or exciton dissociation at the interface of PEDOT:PSS and EML. The energy level difference between PEDOT:PSS and perovskite also hinders hole injection from PEDOT:PSS into EML. Therefore, in order to improve the performance of PeLEDs, it is necessary to reduce the hole injection barrier between PEDOT:PSS and the emission layer and enhance the radiative recombination of carriers in the emission layer [19,20,21]. 

Unbalanced electron and hole injection can reduce device efficiency and operational stability. In many green and blue PeLEDs, the highest occupied molecular orbital (HOMO) of HTLs is much higher than the valence band maximum (VBM) of the perovskite emission layer, resulting in a gap between the HTL and EML. Thus, the hole injection barrier is large and the hole injection efficiency is low. However, the electron injection barrier between the electron transport layer (ETL, such as TPBi) and the perovskite EML is small, and thus the electron injection efficiency is high [22]. Lower hole injection can lead to an imbalance in the carrier injection, resulting in enhanced nonradiative recombination and a higher turn-on voltage. In order to achieve a more balanced carrier injection, a double-layer HTL can be used to improve the hole transport capability [23]. Wang et al. reported a facile method to fabricate green PeLEDs with dual HTLs (TFB/PVK), where the cascaded energy level arrangement of bilayer HTLs facilitated hole injection and charge balance. Because of the reduced non-radiative recombination in perovskite nanocrystals (NCs), the maximum current efficiency (CE) and external quantum efficiency (EQE) of the device are 53.5 cd/A and 12.9%, respectively. The EQE value is about 1.7 times and 3 times that of the device with PVK HTL and TFB HTL, respectively [24]. Wu et al. introduced poly(9-vinylcarbazole) (PVK) into the anti-solvent chloroform, and the introduction of 0.1 mg/mL PVK could promote the formation of smooth and dense perovskite films through surface defect passivation. The photoluminescence quantum efficiency (PLQY) of the perovskite film is as high as 20.70%, and the optimized blue PeLED has a maximum luminance of 3136 cd/m^2^ and a maximum EQE of 3.49% [25]. It can be seen that PVK helps to improve the hole injection efficiency and the quality of perovskite films, showing great application prospects. However, there are still some problems to be studied in terms of process parameters and mechanisms. In this paper, perovskite films were prepared by staged spin coating, and the effects of anti-solvent ethyl acetate and PEDOT:PSS/PVK bilayer HTLs on the optoelectronic properties of MAPbBr_3_ PeLEDs devices were investigated.

## 2. Experimental Section

The device structure prepared in this paper is ITO/PEDOT:PSS (40 nm)/PVK/MAPbBr_3_ (60 nm)/TPBi (30 nm)/LiF (0.5 nm)/Al (120 nm). Methylammonium bromide (CH_3_NH_3_Br, Xi’an Polymer Light Technology Co., Ltd., Xi’an, China) and lead bromide (PbBr_2_, 99.999%, Xi’an Polymer Light Technology Co., Ltd., Xi’an, China) with a molar ratio of 2:1 were dispersed in N,N-Dimethylformamide solvent (DMF, 99.9%, Sigma-Aldrich, Merck KGaA, Darmstadt, Germany) and stirred at 500 rpm and 60 °C for 12 h in a glove box to obtain a perovskite precursor solution (MAPbBr_3_) with a concentration of 5 wt%. Before fabricating PeLEDs, ITO substrates with a sheet resistance of ~15 Ω/m^2^ were sequentially cleaned with acetone, alcohol, and water. After drying with nitrogen, the ITO glass substrates were pretreated with oxygen plasma to increase their work function and optimize the surface. Then, the substrates were transferred to a nitrogen glove box. Forty nanometer PEDOT:PSS (Clevios P AI4083) was spin-coated at 8000 rpm for 30 s on the ITO substrate as a hole injection layer and annealed at 130 °C for 15 min on a constant temperature heating plate. Next, PVK was dissolved in chlorobenzene to prepare PVK solutions with concentrations of 8 and 12 mg/mL, and spin-coated at 2000 rpm for 40 s as a hole transport layer, and annealed at 130 °C for 10 min. Then, the perovskite solution was spin-coated as the emission layer by means of staged spin coating (3000 rpm and 10 s for the first stage, 8000 rpm and 30 s for the second stage), and 200 μL of ethyl acetate was dropped at different times (10 s, 20 s, and 30 s) after the spin coating of the perovskite precursor solution started. The annealing temperature and annealing time of the perovskite layer are 80 °C and 10 min, respectively. Finally, the samples were transferred to a thermal evaporation system for orderly deposition of a 30 nm electron transport layer (TPBi), a 0.5 nm electron injection layer (LiF), and a 120 nm cathode (Al). The effective light-emitting area of each device is about 0.1 cm^2^. All PeLEDs were tested in ambient air without being encapsulated.

In this paper, a stylus profiler (Alpha-Step D-600, KLA Corporation, Milpitas, CA, USA) was used to measure the thickness of functional layers such as HTL and perovskite films. A luminescence spectrometer (HITACHI F-4600, Hitachi Limited, Hitachi, Japan) was used to measure photoluminescence (PL) spectra (excitation wavelength at 315 nm). A scanning electron microscope (FEI Sirion FEG, FEI Corporation, Eindhoven, The Netherlands) and an X-ray diffractometer (Panalytical Empyrean, PANalytical B. V., Almelo, The Netherlands) were used to measure the surface morphology and XRD patterns of the perovskite films. A digital source meter (Keithley 2400, Tektronix, Inc., Beaverton, OR, USA), a digital multimeter (Keithley 2000, Tektronix, Inc., Beaverton, OR, USA), and a silicon photodetector were connected to build a luminance-current-voltage (L-I-V) test system, which is used to detect the photoelectric characteristics of PeLEDs. An optical fiber spectrometer (Ocean Optics USB4000-XR1, Tektronix, Inc., Beaverton, OR, USA) was used to observe the electroluminescence (EL) spectra of PeLEDs.

## 3. Results and Discussion

**Figure 1:**
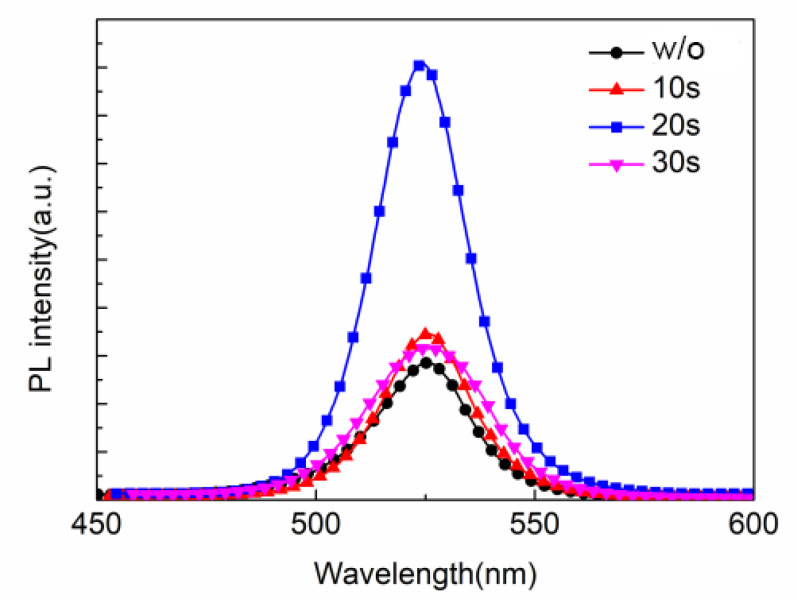
Photoluminescence spectra of perovskite films treated with ethyl acetate dropwise at different times.

Firstly, ITO/PEDOT:PSS (40 nm)/MAPbBr_3_ (60 nm)/TPBi (30 nm)/LiF (0.5 nm)/Al (120 nm) was used as the device structure to study the effect of anti-solvent ethyl acetate on the performance of PeLEDs in order to determine the optimal anti-solvent condition. Figure 1 shows the PL spectra of the perovskite films with ethyl acetate dropped at different times. The excitation wavelength for the PL test is 315 nm. It can be found that all the PL peak wavelengths of the perovskite films are around 525 nm, suggesting that the addition of ethyl acetate does not affect the position of the luminescence peak. As the dropping time of ethyl acetate moved backward, the PL intensity of the perovskite film reached its maximum value when the dropping time was 20 s. When the dropping time of ethyl acetate was further extended to 30 s, the PL intensity of the perovskite film decreased.

**Figure 2:**
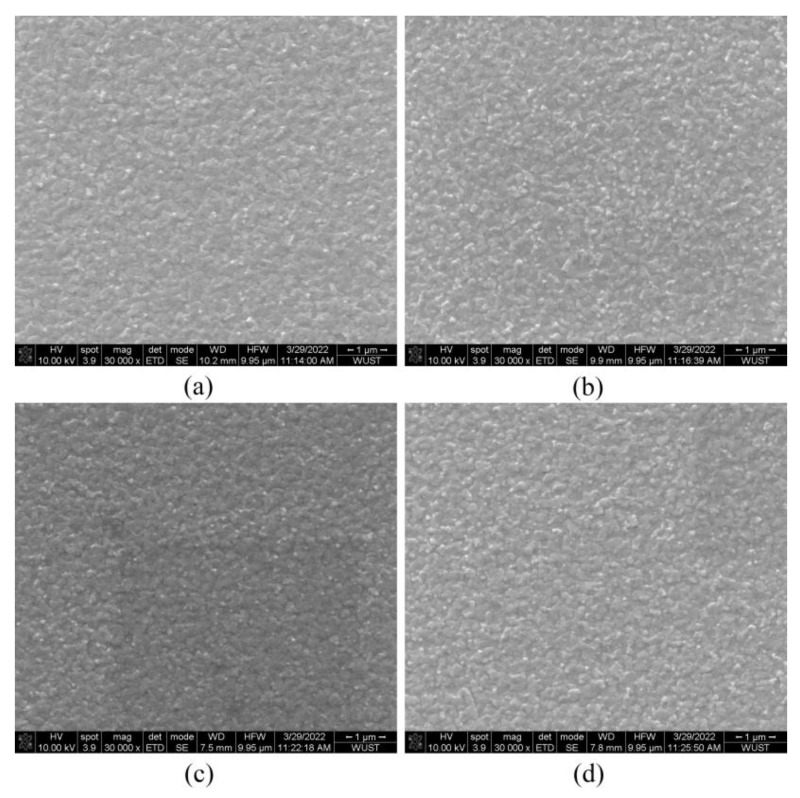
SEM images of perovskite films with ethyl acetate dropped at different times (**a**) without ethyl acetate (**b**) 10 s (**c**) 20 s (**d**) 30 s.

Figure 2 shows the SEM images of the perovskite films. It can be found that the addition of ethyl acetate did not evidently reduce the size of the perovskite particles as expected. When the dropping time is 20 s, a dense perovskite film with uniform particles of slightly reduced size is obtained. At other dropping times, some large perovskite particles were found, suggesting poor film uniformity. The above results show that dropping ethyl acetate can help improve the quality of the perovskite film. When the dropping time of ethyl acetate was 20 s, the PL intensity reached its maximum, and the PL intensity began to decrease when the dropping time of ethyl acetate continued to be delayed. The result is consistent with the SEM images. This is because the dropping time is too late, and thus ethyl acetate could not fully participate in the formation process of perovskite film, resulting in a decrease in the quality of the perovskite film. These results show that when the dropping time of ethyl acetate is 20 s, the density of the film is optimal, and the corresponding PL intensity is the highest.

**Figure 3:**
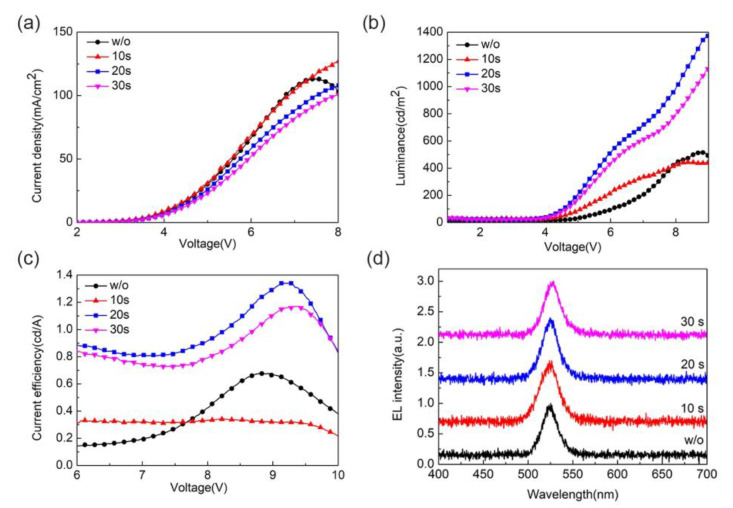
Performance of PeLEDs with ethyl acetate dropped at different times (**a**) current density vs. voltage curve, (**b**) luminance vs. voltage curve, (**c**) current efficiency vs. voltage curve, and (**d**) electroluminescence spectra.

Figure 3 shows the effect of the dropping time of ethyl acetate on the optoelectronic performance of PeLEDs. Table 1 shows the device parameters of PeLEDs with different dropping times of ethyl acetate, including the maximum luminance, maximum current efficiency (CE), and turn-on voltage of the devices. Among them, the control device without ethyl acetate has a maximum brightness of 518 cd/m^2^, a maximum CE of 0.67 cd/A, and a turn-on voltage of 2.56 V. As for the experimental devices, ethyl acetate was dropped at 10 s, 20 s, and 30 s, respectively. When the dropping time of ethyl acetate was 20 s, the optimum performance of the device was obtained, in which the maximum luminance was 1378 cd/m^2^, the maximum CE was 1.34 cd/A, and the turn-on voltage was 2.71 V. When ethyl acetate was dropped for 20 s, the device’s maximum luminance increased by 1.66 times compared to the control device, and its maximum CE increased by 100%. The optoelectronic properties of the devices are well consistent with the PL and SEM data shown in Figure 1 and Figure 2. Ethyl acetate with an appropriate dropping time can promote the formation of uniform and dense perovskite films, in which the PL intensity is also greatly improved, leading to improved luminance and current efficiency. When the dropping time is 30 s, the PL intensity decreases, suggesting decreased device luminance and current efficiency [26]. Figure 3d shows the EL spectra of PeLEDs using ethyl acetate dropped at different times. All the EL spectra were tested at a current density of 50 mA/cm^2^. It can be found that all the EL peak wavelengths of the perovskite films are around 525 nm, indicating that the addition of ethyl acetate does not affect the position of the luminescence peak.

**Figure 4:**
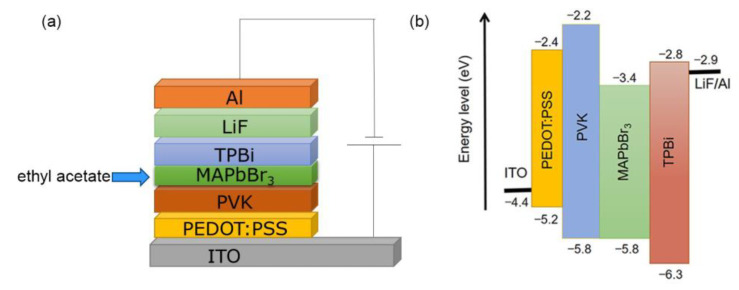
(**a**) Structure and energy (**b**) level diagram of the PeLED device.

After determining the dropping condition of anti-solvent ethyl acetate, the effect of PEDOT:PSS/PVK double-layer HTL on the photoelectric properties of PeLEDs was studied. Figure 4 is a schematic structural diagram and an energy level diagram of the device after inserting PVK between PEDOT:PSS and MAPbBr_3_. The introduction of PVK is expected to reduce the hole injection barrier between the HTL and the perovskite emission layer and thus improve the hole injection capability. The increased electron barrier can block the transfer of electrons from the perovskite layer to the ITO substrate, so that more electrons stay in the emission layer and increase the probability of radiative recombination of carriers, thereby effectively improving the optoelectronic properties of PeLEDs [24,25,27]. 

**Figure 5:**
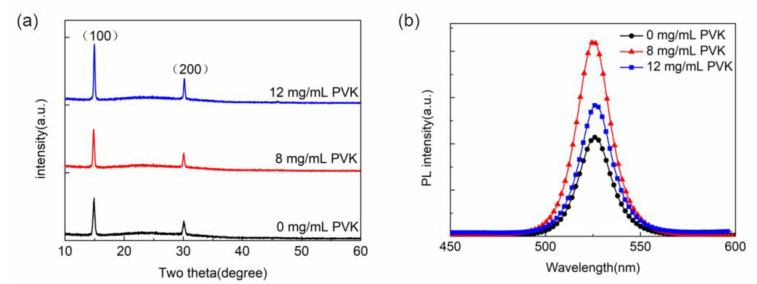
XRD spectra (**a**) and PL spectra (**b**) of perovskite films prepared on PEDOT:PSS/PVK double-layer HTL.

Figure 5a shows the XRD patterns of the perovskite films prepared on the PEDOT:PSS/PVK hole transport layer. It can be seen that all XRD patterns have two characteristic peaks at 15° and 30°, which correspond to the (100) and (200) crystal faces of the perovskite crystal, respectively. After adding PVK, both diffraction peaks are enhanced with the increase in PVK concentration, indicating that the addition of PVK can improve the crystalline quality of perovskite particles. Figure 5b shows the PL spectra of the perovskite films prepared on the PEDOT:PSS/PVK double-layer HTL. It can be found that all the PL peak wavelengths of the perovskite films are around 525 nm, suggesting that the introduction of PVK does not affect the position of the luminescence peak. When the PVK concentration is 8 mg/mL, the PL intensity reaches its largest value. The PL intensity decreases as the PVK concentration is increased to 12 mg/mL, which is also slightly higher than without PVK. These results indicate that an appropriate concentration of PVK can help to suppress the luminescence quenching between PEDOT:PSS and MAPbBr_3_ and enhance the photoluminescence ability of perovskite films.

**Figure 6:**
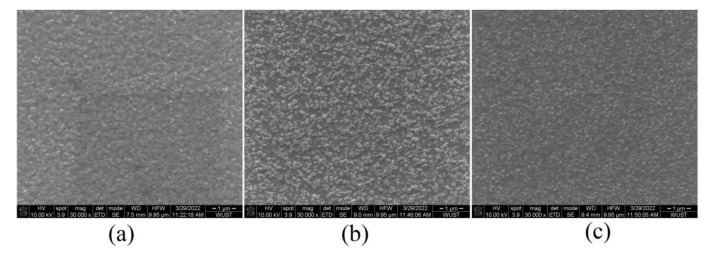
SEM images of perovskite films prepared on PEDOT:PSS/PVK double-layer HTL with (**a**) 0 mg/mL PVK, (**b**) 8 mg/mL PVK, and (**c**) 12 mg/mL PVK.

Figure 6 shows the SEM images of the perovskite films prepared on the PEDOT:PSS/PVK double-layer HTL. When PVK is not added, the perovskite film is relatively dense, and the addition of PVK will significantly reduce the film coverage, which is expected to lead to a decrease in luminescence performance. Meanwhile, the addition of PVK leads to the reduction of perovskite crystal particles, which contributes to the spatial confinement of excitons and the improvement of luminescence properties. These results indicate that the addition of PVK did not optimize the surface coverage of perovskite films but could reduce the particle size. Thus, selecting an appropriate PVK concentration is critical.

**Figure 7:**
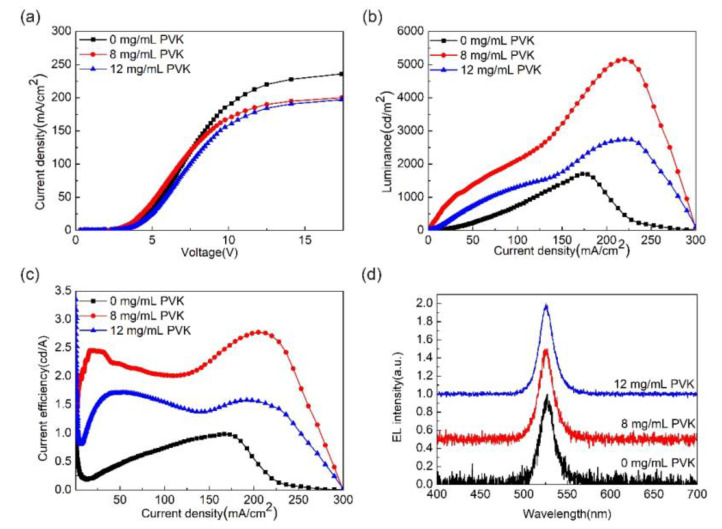
Photoelectric properties of PeLEDs using PEDOT:PSS/PVK bilayer hole transport layer (**a**) current density-voltage curve, (**b**) luminance-current density curve, (**c**) current efficiency-current density curve, and (**d**) electroluminescence spectra.

Figure 7 shows the optoelectronic properties of the PeLEDs using the EDOT:PSS/PVK double-layer HTL, and Table 2 shows the corresponding device parameters. As expected, PVK has a deeper HOMO level (−5.8 eV) compared with PEDOT:PSS (−5.2 eV), which may help to enhance the hole injection. However, in our work, the current density decreased with increasing PVK concentration, which can be explained by the poor conductivity of PVK. The degradation of luminance and current efficiency at high current density may be caused by joule heating or Auger recombination in conjunction with high carrier density, as shown in Figure 7b,c. It can be seen from Table 2 that the control device without PVK has a maximum luminance of 1700 cd/m^2^, a maximum current efficiency (CE) of 0.96 cd/A, and a turn-on voltage of 2.48 V. When the PVK concentration is 8 mg/mL, the maximum luminance of the device is 5139 cd/m^2^, the maximum CE is 2.77 cd/A, and the turn-on voltage is 2.84 V. When the PVK concentration is 12 mg/mL, the device performance is degraded, and the maximum luminance is 2713 cd/m^2^, the maximum CE is 1.72 cd/A, and the turn-on voltage is 2.78 V. The experimental results show that when the PVK concentration is 8 mg/mL, the performance of the device is optimal, the maximum luminance of the device is 2.02 times higher than that of the control device, and the maximum CE is increased by 188%. Figure 7d shows the EL spectra of PeLEDs using EDOT:PSS/PVK hole transport layers. It can be found that all the EL peak wavelengths of the perovskite films are around 525 nm, indicating that the addition of PVK does not affect the position of the luminescence peak. The above results are consistent with the PL results shown in Figure 5b, which can be explained by the following aspects: Firstly, the addition of PVK leads to the reduction of perovskite crystal particles, which contributes to the spatial confinement of excitons, which leads to the improvement of radiative recombination of carriers in the emission layer. Secondly, according to the PL experimental results, 8 mg/mL PVK helps to suppress the luminescence quenching at the interface between PEDOT:PSS and MAPbBr_3_, thereby improving the device performance. However, when the PVK concentration is 12 mg/mL, the device performance is significantly reduced, and the main factor affecting the device performance is the film quality. The SEM results show that the addition of excessive PVK will reduce the surface coverage of the film. Defect states may exist at the interface between PVK and perovskite, and excitons are easily trapped by defects at the interface, resulting in the degradation of the luminescence performance of the device.

## 4. Conclusions

In this paper, the effects of ethyl acetate and the PEDOT:PSS/PVK bilayer hole transport layer on the optoelectronic properties of MAPbBr_3_ PeLEDs were investigated. The introduction of ethyl acetate can facilitate the formation of dense and uniform perovskite films with small particles, thereby improving the luminescence performance of the device. When the PVK concentration was 8 mg/mL, the performance of the device was optimal, with a maximum luminance of 5139 cd/m^2^, and a maximum current efficiency of 2.77 cd/A. Compared with the control device without PVK, the maximum luminance is increased by 2.02 times, and the maximum CE is increased by 188%. The addition of PVK leads to the reduction of perovskite crystal particles, which contributes to the spatial confinement of excitons and improves the probability of radiative recombination of carriers in the emission layer. Meanwhile, PVK helps to suppress the luminescence quenching between PEDOT:PSS and MAPbBr_3_, thereby enhancing the photoelectric properties of PeLEDs. However, adding excessive PVK reduces the film surface coverage, and higher concentrations of PVK lead to the degradation of device performance. The results of this paper can provide a basis for the improvement and industrialization of PeLEDs.

## Figures and Tables

**Table 1:** Summary of the device parameters of PeLEDs with different dropping times of ethyl acetate.

Dropping Time	L_max_ (cd/m^2^)	CE_max_ (cd/A)	Turn-On Voltage (V)
w/o	518	0.67	2.56
10 s	441	0.34	2.66
20 s	1378	1.34	2.71
30 s	1165	1.17	2.76

**Table 2:** Summary of the device parameters of PeLEDs with different concentrations of PVK.

PVK	L_max_ (cd/m^2^)	CE_max_ (cd/A)	Turn-On Voltage (V)
0 mg/mL	1700	0.96	2.48
8 mg/mL	5139	2.77	2.84
12 mg/mL	2713	1.72	2.78

## Data Availability

Not applicable.

## References

[1] Tang C.W., VanSlyke S.A. (1987). Organic electroluminescent diodes. Appl. Phys. Lett..

[2] Liu N., Shi W.X., Zhou Y.M., Cao X.A. (2019). Impact of dopant aggregation on the EL of blue fluorescent host-dopant emitters. IEEE Electron Device Lett..

[3] Shi W.X., Liu N., Zhou Y.M., Cao X.A. (2019). Effects of postannealing on the characteristics and reliability of polyfluorene organic light-emitting diodes. IEEE Trans. Electron Devices.

[4] Liu N., Mei S.J., Sun D.W., Shi W.X., Feng J.H., Zhou Y.M., Mei F., Xu J.X., Jiang Y., Cao X.A. (2019). Effects of charge transport materials on blue fluorescent organic light-emitting diodes with a host-dopant system. Micromachines.

[5] Liu L., Li S., Zhou Y.M., Liu L.Y., Cao X.A. (2017). High-current stressing of organic light-emitting diodes with different electron-transport materials. Microelectron. Reliab..

[6] Wang Z., Cheng T., Wang F., Dai S., Tan Z.A. (2016). Morphology Engineering for High-Performance and Multicolored Perovskite Light-Emitting Diodes with Simple Device Structures. Small.

[7] Li Z., Yang M., Park J.S., Wei S.H., Berry J.J., Zhu K. (2016). Stabilizing perovskite structures by tuning tolerance factor: Formation of formamidinium and cesium lead iodide solid-state alloys. Chem. Mater..

[8] Noh J.H., Im S.H., Heo J.H., Mandal T.N., Seok S.I. (2013). Chemical management for colorful, efficient, and stable inorganic–organic hybrid nanostructured solar cells. Nano Lett..

[9] Xing G., Mathews N., Lim S.S., Yantara N., Liu X., Sabba D., Grätzel M., Mhaisalkar S., Sum T.C. (2014). Low-temperature solution-processed wavelength-tunable perovskites for lasing. Nat. Mater..

[10] Veldhuis S.A., Boix P.P., Yantara N., Li M., Sum T.C., Mathews N., Mhaisalkar S.G. (2016). Perovskite materials for light-emitting diodes and lasers. Adv. Mater..

[11] Zhou Y.M., Mei S.J., Sun D.W., Liu N., Mei F., Xu J.X., Cao X.A. (2019). Improved charge injection and transport of light-emitting diodes based on two-dimensional materials. Appl. Sci..

[12] Sun D.W., Zhang K., Mei S.J., Xu J.X., Jiang Y., Xiao X.H., Zhou Y.M., Mei F. (2021). High performance perovskite LEDs via SPR and enhanced hole injection by incorporated MoS_2_. J. Phys. D Appl. Phys..

[13] Zhou Y.M., Mei S.J., Feng J.J., Sun D.W., Mei F., Xu J.X., Cao X.A. (2020). Effects of PEDOT:PSS:GO composite hole transport layer on the luminescence of perovskite light-emitting diodes. Rsc Adv..

[14] Zhou Y.M., Mei S.J., Sun D.W., Liu N., Shi W.X., Feng J.H., Mei F., Xu J.X., Jiang Y., Cao X.A. (2019). Improved efficiency of perovskite light-emitting diodes using a three-step spin-coated CH_3_NH_3_PbBr_3_ emitter and a PEDOT:PSS/MoO_3_-ammonia composite hole transport layer. Micromachines.

[15] Dong Q., Fang Y., Shao Y., Mulligan P., Qiu J., Cao L., Huang J. (2015). Electron-hole diffusion lengths > 175μm in solution-grown CH_3_NH_3_PbI_3_ single crystals. Science.

[16] Kim Y.H., Cho H., Heo J.H., Kim T.S., Myoung N., Lee C.L., Im S.H., Lee T.W. (2015). Multicolored organic/inorganic hybrid perovskite light-emitting diodes. Adv. Mater..

[17] Huang J., Yuan Y., Shao Y., Yan Y. (2017). Understanding the physical properties of hybrid perovskites for photovoltaic applications. Nat. Rev. Mater..

[18] Kumawat N.K., Gupta D., Kabra D. (2017). Recent Advances in Metal Halide-Based Perovskite Light-Emitting Diodes. Energy Technol..

[19] Cho H., Jeong S.H., Park M.H., Kim Y.H., Wolf C., Lee C.L., Heo J.H., Sadhanala A., Myoung N., Yoo S. (2015). Overcoming the electroluminescence efficiency limitations of perovskite light-emitting diodes. Science.

[20] Miao Y., Cheng L., Zou W., Gu L., Zhang J., Guo Q., Peng Q., Xu M., He Y., Zhang S. (2020). Microcavity top-emission perovskite light-emitting diodes. Light Sci. Appl..

[21] Wang A., Guo Y., Muhammad F., Deng Z. (2017). Controlled Synthesis of Lead-Free Cesium Tin Halide Perovskite Cubic Nanocages with High Stability. Chem. Mater..

[22] Hao F., Stoumpos C.C., Chang RP H., Kanatzidis M.G. (2014). Anomalous Band Gap Behavior in Mixed Sn and Pb Perovskites Enables Broadening of Absorption Spectrum in Solar Cells. J. Am. Chem. Soc..

[23] Dai X., Zhang Z., Jin Y., Niu Y., Cao H., Liang X., Chen L., Wang J., Peng X. (2014). Solution-processed, high-performance light-emitting diodes based on quantum dots. Nature.

[24] Wang W., Wu Z., Ye T., Ding S., Wang K., Peng Z., Sun X.W. (2021). High-performance perovskite light-emitting diodes based on double hole transport layers. J. Mater. Chem. C.

[25] Wu Y., Yuan F., Fu F., Liu C., Jiao B., Zhang F., Wu Z. (2021). Enhanced performance of spectra stable blue perovskite light-emitting diodes through Poly (9-vinylcarbazole) interlayer incorporation. Org. Electron..

[26] Li J., Xu L., Wang T., Song J., Chen J., Xue J., Dong Y., Cai B., Shan Q., Han B. (2017). 50-Fold EQE improvement up to 6.27% of solution-processed all-inorganic perovskite CsPbBr_3_ QLEDs via surface ligand density control. Adv. Mater..

[27] Lan L., Liu B., Tao H., Zou J., Jiang C., Xu M., Wang L., Peng J., Cao Y. (2019). Preparation of efficient quantum dot light-emitting diodes by balancing charge injection and sensitizing emitting layer with phosphorescent dye. J. Mater. Chem. C.

